# Purinergic and Cholinergic Drugs Mediate Hyperventilation in Zebrafish: Evidence from a Novel Chemical Screen

**DOI:** 10.1371/journal.pone.0154261

**Published:** 2016-04-21

**Authors:** Saman Rahbar, Wen Pan, Michael G. Jonz

**Affiliations:** Department of Biology, University of Ottawa, Ottawa, Ontario, Canada; Institute of Hydrobiology, Chinese Academy of Sciences, CHINA

## Abstract

A rapid test to identify drugs that affect autonomic responses to hypoxia holds therapeutic and ecologic value. The zebrafish (*Danio rerio*) is a convenient animal model for investigating peripheral O_2_ chemoreceptors and respiratory reflexes in vertebrates; however, the neurotransmitters and receptors involved in this process are not adequately defined. The goals of the present study were to demonstrate purinergic and cholinergic control of the hyperventilatory response to hypoxia in zebrafish, and to develop a procedure for screening of neurochemicals that affect respiration. Zebrafish larvae were screened in multi-well plates for sensitivity to the cholinergic receptor agonist, nicotine, and antagonist, atropine; and to the purinergic receptor antagonists, suramin and A-317491. Nicotine increased ventilation frequency (*f*_V_) maximally at 100 μM (EC_50_ = 24.5 μM). Hypoxia elevated *f*_V_ from 93.8 to 145.3 breaths min^-1^. Atropine reduced the hypoxic response only at 100 μM. Suramin and A-317491 maximally reduced *f*_V_ at 50 μM (EC_50_ = 30.4 and 10.8 μM) and abolished the hyperventilatory response to hypoxia. Purinergic P2X3 receptors were identified in neurons and O_2_-chemosensory neuroepithelial cells of the gills using immunohistochemistry and confocal microscopy. These studies suggest a role for purinergic and nicotinic receptors in O_2_ sensing in fish and implicate ATP and acetylcholine in excitatory neurotransmission, as in the mammalian carotid body. We demonstrate a rapid approach for screening neuroactive chemicals in zebrafish with implications for respiratory medicine and carotid body disease in humans; as well as for preservation of aquatic ecosystems.

## Introduction

The ability to maintain internal oxygen (O_2_) levels within an optimal physiological range is essential for development and survival in vertebrates as this facilitates O_2_ delivery to cells and matching of metabolic demands. Vertebrates have respiratory chemoreceptors—specialized cells that sense changes in O_2_ partial pressure (*P*_O2_)—which elicit compensatory cardioventilatory adjustments during periods of hypoxia. In mammals, the main peripheral chemoreceptors are type I cells of the carotid body, which store neurotransmitters that are released into a chemical synapse during chemoexcitation [1–5]. Although controversial, there is compelling evidence that the primary excitatory neurotransmitters, which activate afferent nerve terminals in the carotid body, include acetylcholine (ACh) and 5’-adenosine triphosphate (ATP) [6–12]. ACh and ATP activate nicotinic and purinergic P2X receptors, respectively, both of which are non-selective cation channels that mediate excitation.

Homologous to carotid body type I cells are the neuroepithelial cells (NECs) in the gills of teleost fish [13–16]. These cells respond to a decrease in *P*_O2_ with K^+^ channel inhibition and membrane depolarization [17,18]; and NECs are believed to initiate reflex responses to hypoxia, such as hyperventilation, increased ventilatory amplitude and bradycardia [15,16,19,20]. It is predicted that during hypoxic stimulation NECs release neurotransmitters into a chemical synapse that activate afferent nerve fibres within the gill filaments. NECs contain synaptic vesicles [13,14] endowed with neurotransmitters, including serotonin (5-hydroxytryptamine, 5-HT), acetylcholine (ACh) and nitric oxide [16,21–24]; and NECs undergo dynamic changes in intracellular Ca^2+^ upon stimulation [25–27]. However, unlike the carotid body, little is known about cholinergic activity in the fish gill; and there is no functional evidence for purinergic receptors in O_2_ sensing in fish.

We hypothesized that exogenous application of neurochemicals that target purinergic and cholinergic receptors accordingly modify the hyperventilatory response to hypoxia via chemosensory pathways in the gills. Zebrafish were used as a model organism as they are a convenient size for recording changes in ventilatory frequency, and rapidly develop a mature O_2_-chemosensory system in the gills during the first two weeks of development [23,28]. We demonstrate purinergic and cholinergic control of the hyperventilatory response to hypoxia in zebrafish, thereby highlighting the evolutionary significance of these mechanisms in O_2_ sensing in vertebrates. Numerous studies have used zebrafish to develop *in vivo* chemical screens for drug discovery [29,30]. As an additional objective, we have generated a rapid procedure for the screening of chemicals that may be studied further for their potential as therapeutic agents for respiratory disorders in humans; and for the identification of pharmaceutical or industrial products that threaten aquatic ecosystems.

## Materials and Methods

### Ethical statement

Handling and care of animals were conducted in accordance with the Canadian Council on Animal Care (CCAC) and all protocols were approved by the Animal Care Committee at the University of Ottawa (protocol no. BL-1760). Behavioural experiments were performed under MS-222 (tricaine) anesthesia and all efforts were made to minimize stress on the animals.

### Animals

Wild-type zebrafish (*Danio rerio*) were obtained from a commercial supplier (Mirdo Importations Inc., Montreal, Canada) and held in a closed, re-circulated facility at the University of Ottawa. They were maintained at 28.5°C on a 14:10 hour light:dark cycle. Adults were bred using breeding traps as per methods described by Westerfield [31]. In brief, the resulting embryos were kept in Petri dishes containing E3 embryo medium (5 mM NaCl, 0.17 mM KCl, 0.33 mM CaCl_2_ 2H_2_O, 0.33 mM MgSO_4_ 7H_2_O and 0.3 mg l^-1^ of methylene blue at pH 7.8) and incubated at 28.5°C for 5 days. At 5 days post fertilization (dpf), larvae were transferred to small tanks filled with treated system water (0.3 mg l^-1^ with methylene blue).

### Behavioural assays

The larvae used in this study ranged from 14–16 dpf. At this age, zebrafish have fully innervated gill NECs and mature respiratory lamellae [28,32]. Therefore, physiological responses to neurochemicals can be attributed to branchial rather than extrabranchial (i.e. cutaneous) mechanisms. Larvae were lightly anaesthetized with tricaine (MS-222, Aqualife TMS, Syndel Laboratories, Vancouver, Canada) at 0.04 mg/ml for 2 min and transferred individually into the wells of an opaque, flat-bottomed polystyrene multi-well plate (96-well, Greiner Cellstar, Sigma-Aldrich, Oakville, ON, Canada; S1 Fig, S2 Fig). Up to 8 larvae were tested during each trial; and each well contained 1 larva. Each well was previously filled with Sylgard (Dow Corning Corporation, Midland, MI, USA) that was left overnight to solidify. This reduced the volume of medium in each well to approximately 100 μl and improved observation of fish by reducing movement out of the range of focus of the microscope. Since larvae exhibit a photomotor response when exposed to light during behavioural assays [33], 50 μl of 1% methylcellulose and 0.04 mg/ml tricaine was added to each well to minimize movement. Addition of methylcellulose also allowed us to reduce the concentration of MS 222, compared to previous studies [28]. Fish were allowed to adapt to their new environment for 2 min before any observations were made. Normoxic ventilation rate was then obtained and served as a baseline measurement of ventilation frequency (*f*_V_). The remaining 50 μl of each well was then filled with the treatment drug plus 0.04 mg/ml tricaine or tricaine alone (as the positive control group exposed to hypoxia only). If a stimulatory drug was added, *f*_V_ was measured after an additional 2 min. If an inhibitory drug was applied, the plate was placed in a hypoxic incubator (Forma 3110, ThermoFisher Scientific, Ottawa, ON, Canada) where 100% N_2_ was injected to reduce O_2_ levels to 1% (8 mmHg). *P*_O2_ was measured and stabilized with a thermal conductivity O_2_ sensor. Fish were incubated for 7 min at 28.5°C and *f*_V_ was recorded immediately after. Preliminary experiments had established that an incubation time of 7 min was sufficient to produce hypoxia within the wells and initiate a maximal *f*_V_ response (S3 Fig). For recovery, each larva was transferred to a new well in normal solution plus 0.04 mg/ml tricaine, and *f*_V_ was recorded after 2 min. For all treatment groups, *f*_V_ was recorded by placing the multi-well plate upon the stage of a stereomicroscope (M6, Leica, Wetzlar, Germany) and capturing 10-s videos using Leica Application Suite. Videos were analyzed *post hoc* and *f*_V_ was calculated. All sample sizes are indicated in the figure legends.

### Drugs

For each drug, a range of concentrations (see Results and figure legends) was tested in order to determine the concentration that elicited a half-maximal *f*_V_ response (EC_50_ value). Serotonin (5-HT) is a major neurotransmitter of gill NECs and was previously shown to increase *f*_V_ in larval zebrafish as early as 7 dpf [23]. 5-HT (cat. no. H9523) was thus used to generate a standard *f*_V_ response and to substantiate the present new procedures for behavioural assay and exogenous drug application. Nicotine (cat. no. N5260), a nicotinic ACh receptor agonist, and atropine (cat. no. A0257), a muscarinic ACh receptor antagonist, were used to target cholinergic receptors. In order to examine the role of purinergic receptors in O_2_ sensing, suramin (cat. no. S2671), a broad-spectrum antagonist of P2 receptors, and A-317491 (cat. no. A2979), a novel P2X3- and P2X2/3-specific antagonist, were used. These drugs were obtained from Sigma-Aldrich Corp. (St. Louis, MO, USA). The ATP analogue and purinergic P2 receptor agonist, 2-methylthioadenosine 5'-triphosphate (2-MeSATP), was also tested and was obtained from Tocris Bioscience (cat. no. 1062; Bristol, UK). All drugs were dissolved in water.

### Data analysis and statistics

The D'Agostino-Pearson test for normality was used to determine whether the data collected during behavioural assays conformed to a Gaussian distribution. Repeated measures analysis of variance (ANOVA) was used to compare *f*_V_ between control, treatment and recovery in all experiments. A Bonferroni *post hoc* test was then used to determine whether mean *f*_V_ was significantly different between control and treatment, and between recovery and control groups (*P* < 0.05).

Curves for dose vs. response were prepared to determine the effective concentration of each drug that gave a half-maximal *f*_V_ response (EC_50_). For excitatory drugs (i.e. 5-HT and nicotine), the response ratio for each individual to each drug concentration was first calculated by dividing treatment *f*_V_ by control *f*_V_. This procedure was necessary because basal *f*_V_ varied between trials. The response ratio therefore presented a clear measure of the response within each trial relative to control. Means of these ratios from each group were then further normalized to percent of maximum *f*_V_ by the following: maximum *f*_V_ was taken as the highest response ratio within each group, and all other points within that group were divided by this value and converted to percentage. For inhibitory drugs (i.e. suramin and A-317491) that were co-administered with hypoxia, *f*_V_ was suppressed at high concentrations. For these experiments, dose vs. percent maximum inhibition was plotted to calculate EC_50_. The response ratio for each drug was calculated as above (i.e. treatment *f*_V_ divided by control *f*_V_). Maximum inhibition was taken as the concentration with the lowest response ratio, and this was then divided by other points at each concentration and converted to percentage. For calculation of EC_50_ in all graphs, a line was fit to the data using a one-phase exponential association model (Prism v5.0, GraphPad Software Inc., La Jolla, CA, USA) with least squares and constrained to a *y*_max_ of 100 following the equation *y* = *y*_max_ (1-e^-*kx*^).

### Immunohistochemistry and confocal microscopy

Zebrafish larvae at 16 dpf were prepared for immunohistochemistry following previously established procedures [23,28]. Zebrafish were euthanized with 1 mg ml^-1^ MS-222. Whole larvae were then fixed by immersion in a phosphate-buffered solution (PBS; 137 mM NaCl, 15.2 mM Na_2_HPO_4_, 2.7 mM KCl, 1.5 mM KH_2_PO_4_ at pH 7.8, [14]) containing 4% paraformaldehyde at 4°C overnight. After rinsing, whole larvae were permeabilized for 24–48 h using 2% Triton X-100 in PBS at 4°C.

Larvae were placed in a solution of primary antibodies, containing polyclonal rabbit anti-5-HT (1:250; cat. no. S5545, Sigma-Aldrich; Antibody ID: AB_477522) and polyclonal guinea pig anti-P2X3 (1:400−1:1,000; cat. no. GP10108; Neuromics, Edina, MN, USA; Antibody ID: AB_2283325), and diluted in 0.5% Triton X-100 at 4°C for 24 h. Tissue was then rinsed in PBS and co-treated with secondary antibodies, anti-rabbit fluorescein isothiocyanate (FITC, 1:50; cat no. 111-095-003, Jackson Immuno Research Laboratories, West Grove, PA, USA) and anti-guinea pig Alexa 594 (1:400; cat. no. A11076, Invitrogen), at room temperature for 1–2 h in the dark. Larvae were rinsed and gill complexes removed and placed on glass slides for imaging.

Polyclonal antibodies for 5-HT were raised in rabbit against a creatinine sulphate complex conjugated with BSA (manufacturer specifications). This antibody has been used to characterize the serotonergic system of gill NECs in several teleost species, including zebrafish [14,23,34]. Antibodies for P2X3 receptors were cloned from rat dorsal root ganglia and recognize residues 383–397 of the carboxy terminus of the receptor. For controls, the P2X3 antibody was pre-treated with a specific blocking peptide (1:100; cat. no. P10108, Neuromics) for 48 h at 4°C before application of the antibody to the tissue for 2 h at room temperature. This procedure eliminated P2X3 immunolabelling.

Tissues stained with fluorescent markers were examined using an upright Nikon A1R microscope with a Nikon Apo 25× water immersion objective lens and a confocal scanning system equipped with Argon (Ar) and Sapphire 561 lasers with peak outputs of 488 nm and 561 nm, respectively. Images were collected using the microscope imaging software NIS Elements 4.13 (Nikon, Tokyo, Japan). Tissues with two fluorescent markers were sequentially scanned with Ar and Sapphire 561 lasers. Images were scanned in optical sections that were 1.5 μm apart for up to 40 sections per z-stack.

## Results

### Verification of procedures for measurement of ventilation frequency following hypoxia or drug treatment

**Fig 1:**
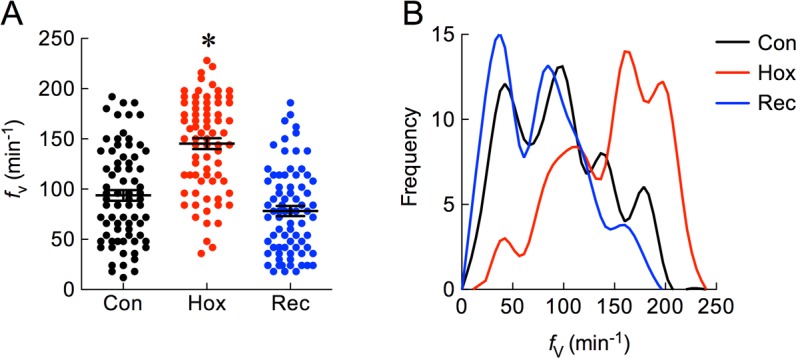
Zebrafish larvae hyperventilated following exposure to severe hypoxia in multi-well plates. (A) Mean ± s.e.m. baseline ventilation frequency (*f*_V_, in min^-1^) was first measured in normoxic controls (Con). Application of hypoxia (Hox) increased *f*_V_, and this was reduced upon recovery (Rec) in normoxic solution. Asterisk indicates a significant difference from control (*n* = 75, *P* < 0.05, repeated measures ANOVA-Bonferroni). (B) A frequency histogram of the data from A indicates that the distribution from all 3 groups was Gaussian.

We first established the ventilatory response to hypoxia, in which zebrafish larvae were exposed to a *P*_O2_ of 8 mmHg for 7 min in muti-well plates. Basal ventilation frequency (*f*_V_) in control fish was recorded at 93.8 ± 5.4 min^-1^ and this increased significantly to 145.3 ± 5.4 min^-1^ (a 1.5-fold change) immediately following hypoxic exposure (Fig 1A; *P* < 0.05, ANOVA-Bonferroni). *f*_V_ returned to just below basal levels (78.2 ± 5.0 min^-1^) during the recovery period. Tests for normality using D'Agostino-Pearson analysis indicated that data from all 3 groups conformed to a normal distribution (Fig 1B; *P* > 0.05).

**Fig 2:**
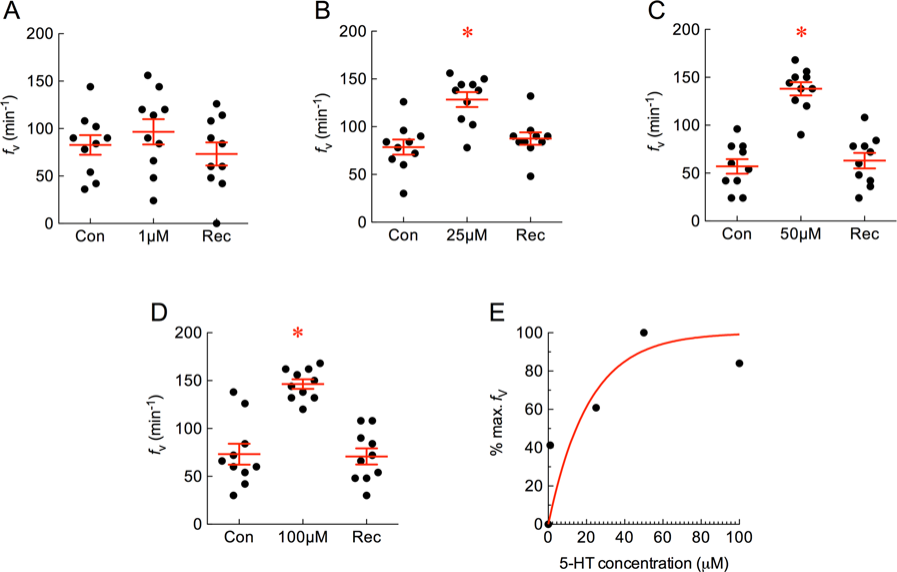
Serotonin (5-hydroxytryptamin, 5-HT) increased mean ± s.e.m. ventilation frequency (*f*_V_, in min^-1^) in a dose-dependent manner. (A−D) Baseline *f*_V_ was measured as control (Con) and 1, 25, 50 or 100 μM 5-HT was applied. Normal solution was replaced during recovery (Rec). Asterisks indicate a significant difference from control (*n* = 10 for each panel; *P* < 0.05, repeated measures ANOVA-Bonferroni). (E) Plot of dose vs. percent of maximum *f*_V_ response. A non-linear one-phase association model with least squares fit indicates an effective half-maximal concentration (EC_50_) of 14.6 μM for 5-HT.

Serotonin (5-HT) is a major neurotransmitter in gill NECs and stimulates chemosensory pathways in the gill to increase *f*_V_ in fish [23,35,36]. We used 5-HT in a range of concentrations as a positive control to demonstrate dose-dependent effects of exogenous drug application on *f*_V_. 5-HT did not have an effect on *f*_V_ at 1 μM, but significantly increased *f*_V_ at 25, 50 and 100 μM (Fig 2A–2D; *P* < 0.05, ANOVA-Bonferroni). The response at 50 μM was taken as maximum *f*_V_, where an *f*_V_ of 138 ± 6.9 min^-1^ was 2.4-fold greater than control. The normalized responses at the concentrations tested were plotted as a percentage of maximum *f*_V_ and indicated an EC_50_ of 14.6 μM (Fig 2E).

### Effects of cholinergic drugs on ventilation frequency

**Fig 3:**
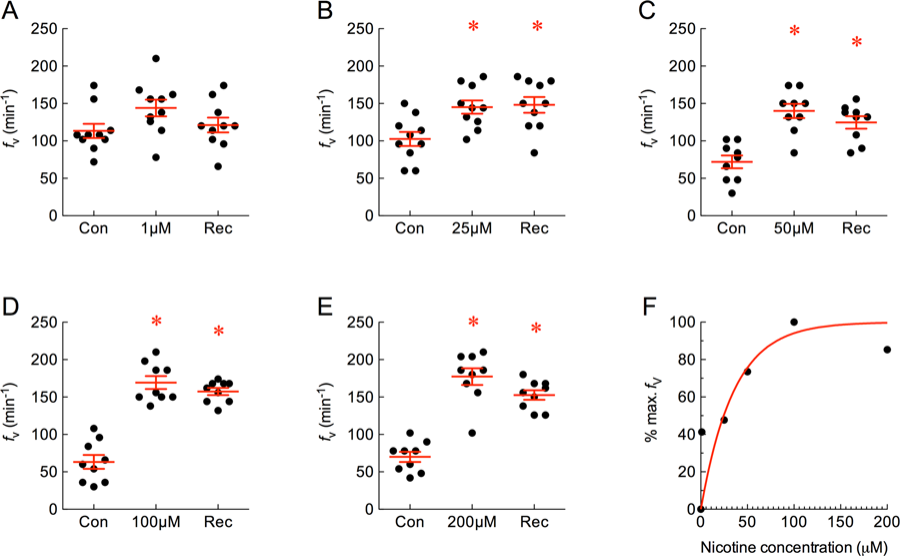
Nicotine increased mean ± s.e.m. ventilation frequency (*f*_V_, in min^-1^) in a dose-dependent manner. (A−E) Baseline *f*_V_ was measured as control (Con) and 1, 25, 50, 100 or 200 μM nicotine was applied. Normal solution was replaced during recovery (Rec). Asterisks indicate a significant difference from control (*n* = 10, 10, 9, 9 and 9; *P* < 0.05, repeated measures ANOVA-Bonferroni). Note that recovery was incomplete at higher concentrations. (F) Plot of dose vs. percent of maximum *f*_V_ response. A non-linear one-phase association model with least squares fit indicates an effective half-maximal concentration (EC_50_) of 24.5 μM for nicotine.

Administration of nicotine over a range of concentrations demonstrated a significant dose-dependent effect on *f*_V_. 1 μM nicotine had only a marginal effect, but at 25, 50, 100 and 200 μM nicotine significantly increased *f*_V_ (Fig 3A–3E; *P* < 0.05, ANOVA-Bonferroni). The response at 100 μM was taken as maximum *f*_V_, where an *f*_V_ of 169.3 ± 8.6 min^-1^ was 2.7-fold greater than control. Note that in these experiments *f*_V_ did not return to basal levels during the recovery period. The normalized responses for nicotine at all concentrations were plotted as a percentage of maximum *f*_V_ and indicated an EC_50_ of 24.5 μM (Fig 3F).

**Fig 4:**
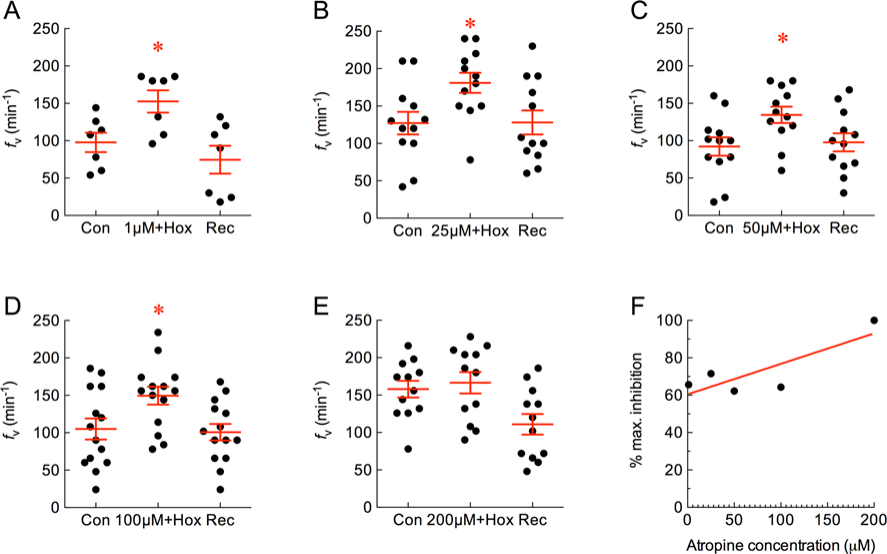
Atropine attenuated the increase in mean ± s.e.m. ventilation frequency (*f*_V_, in min^-1^) induced by hypoxia at high concentration. (A−E) Baseline *f*_V_ was measured as control (Con) and 1, 25, 50, 100 or 200 μM atropine was co-applied with hypoxia. Normal solution was replaced during recovery (Rec). Asterisks indicate a significant difference from control (*n* = 7, 12, 12, 14 and 12; *P* < 0.05, repeated measures ANOVA-Bonferroni). (F) Plot of dose vs. percent inhibition of maximum *f*_V_. A linear regression model with least squares fit indicated no significant effect of atropine (*R*^2^ = 0.68, *P* > 0.05).

The muscarinic antagonist, atropine, was co-applied with hypoxia but did not inhibit the hyperventilatory response at 1, 25, 50 or 100 μM (Fig 4A–4D). *f*_V_ continued to increase significantly in response to hypoxia (*P* < 0.05, ANOVA-Bonferroni) by a factor of 1.4- to 1.5-fold over control values, and this was comparable to the *f*_V_ response to hypoxia alone (Fig 1A). 200 μM atropine, however, abolished the *f*_V_ response to hypoxia (Fig 4E; *P* > 0.05, ANOVA-Bonferroni). The results of the atropine experiments are summarized in Fig 4F. Since atropine did not have a significant effect on inhibiting *f*_V_ at most of the concentrations tested, a dose-response model was not generated to estimate EC_50_.

### Effects of purinergic drugs on ventilation frequency

**Fig 5:**
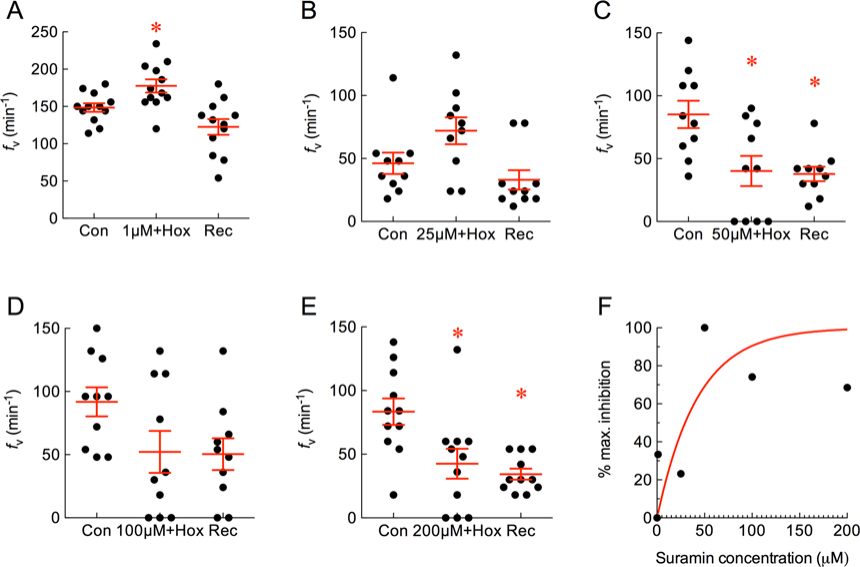
Suramin attenuated the increase in mean ± s.e.m. ventilation frequency (*f*_V_, in min^-1^) induced by hypoxia, and suppressed *f*_V_ below basal levels at high concentrations. (A−E) Baseline *f*_V_ was measured as control (Con) and 1, 25, 50, 100 or 200 μM suramin was co-applied with hypoxia. Normal solution was replaced during recovery (Rec). Asterisks indicate a significant difference from control (*n* = 12, 10, 10, 10 and 11; *P* < 0.05, repeated measures ANOVA-Bonferroni). Note that in (A) hypoxia increased *f*_V_, but in other panels suramin suppressed *f*_V_ and at some concentrations *f*_V_ did not recover. (F) Plot of dose vs. percent inhibition of maximum *f*_V_. A non-linear one-phase association model with least squares fit indicates an effective half-maximal concentration (EC_50_) of 30.4 μM for suramin.

Suramin, a broad-spectrum purinergic antagonist, was co-administered with hypoxia. At 1 μM, suramin had little or no effect, and *f*_V_ increased significantly as a result of hypoxia (Fig 5A; *P* < 0.05, ANOVA-Bonferroni). At 25 μM, suramin reduced the hyperventilatory response to hypoxia such that *f*_V_ did not increase significantly above control (Fig 5B; *P* > 0.05, ANOVA-Bonferroni). When suramin was applied with hypoxia at 50, 100 and 200 μM, *f*_V_ was suppressed significantly below basal levels and did not increase during the recovery period (Fig 5C–5E; *P* < 0.05, ANOVA-Bonferroni). The effect of suramin at 50 μM was taken as maximum inhibition, where an *f*_V_ of 40.2 ± 12 min^-1^ was 2.1-fold lower than control values. The normalized responses at all concentrations were plotted as a percentage of maximum inhibition and indicated an EC_50_ of 30.4 μM (Fig 5F).

**Fig 6:**
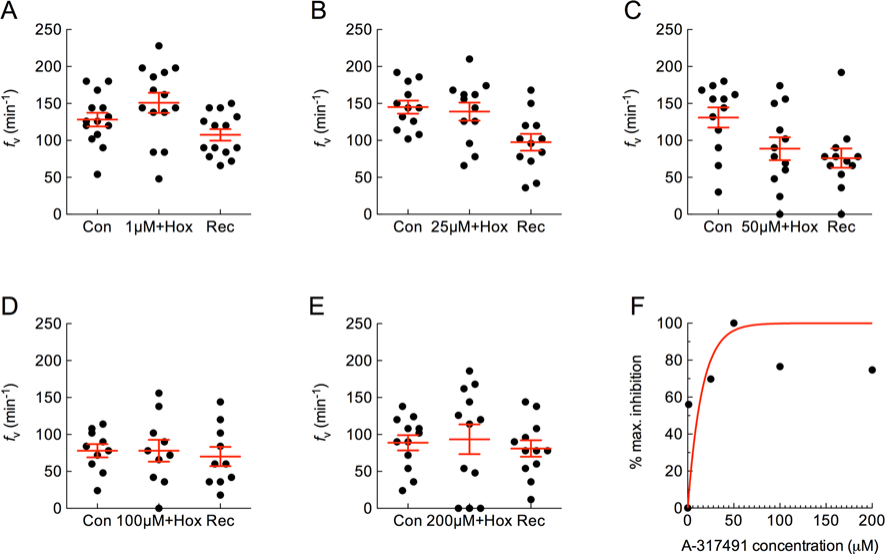
A-317491 attenuated the increase in mean ± s.e.m. ventilation frequency (*f*_V_, in min^-1^) induced by hypoxia. (A−E) Baseline *f*_V_ was measured as control (Con) and 1, 25, 50, 100 or 200 μM A-317491 was co-applied with hypoxia. Normal solution was replaced during recovery (Rec). Treatment groups did not differ from controls (*n* = 14, 12, 12, 10 and 12; *P* > 0.05, repeated measures ANOVA-Bonferroni). (F) Plot of dose vs. percent inhibition of maximum *f*_V_. A non-linear one-phase association model with least squares fit indicates an effective half-maximal concentration (EC_50_) of 10.8 μM for A-317491.

A specific P2X3 blocker, A-317491, was administered with hypoxia. In the presence of the drug at all concentrations ranging from 1−200 μM, hypoxia failed to increase *f*_V_ (Fig 6A–6E; *P* > 0.05, ANOVA-Bonferroni). The effect of A-317491 at 50 μM was taken as maximum inhibition, where an *f*_V_ of 88.8 ± 15 min^-1^ was 1.5-fold lower than control values. The normalized responses at all concentrations were plotted as a percentage of maximum inhibition and indicated an EC_50_ of 10.8 μM (Fig 6F).

The ATP analogue and P2 agonist, 2-MeSATP, was administered to larvae at concentrations from 1–200 μM (S4 Fig). At 100 μM, 2-MeSATP significantly increased *f*_V_ from 70.1 ± 15.4 to 116.0 ± 12.1 min^-1^, a factor of 1.6 (S4 Fig; *P* < 0.05, ANOVA-Bonferroni). 2-MeSATP produced a marginal increase in *f*_V_ at 50 and 200 μM, but was without effect at lower concentrations.

### Localization of P2X3 receptors

**Fig 7:**
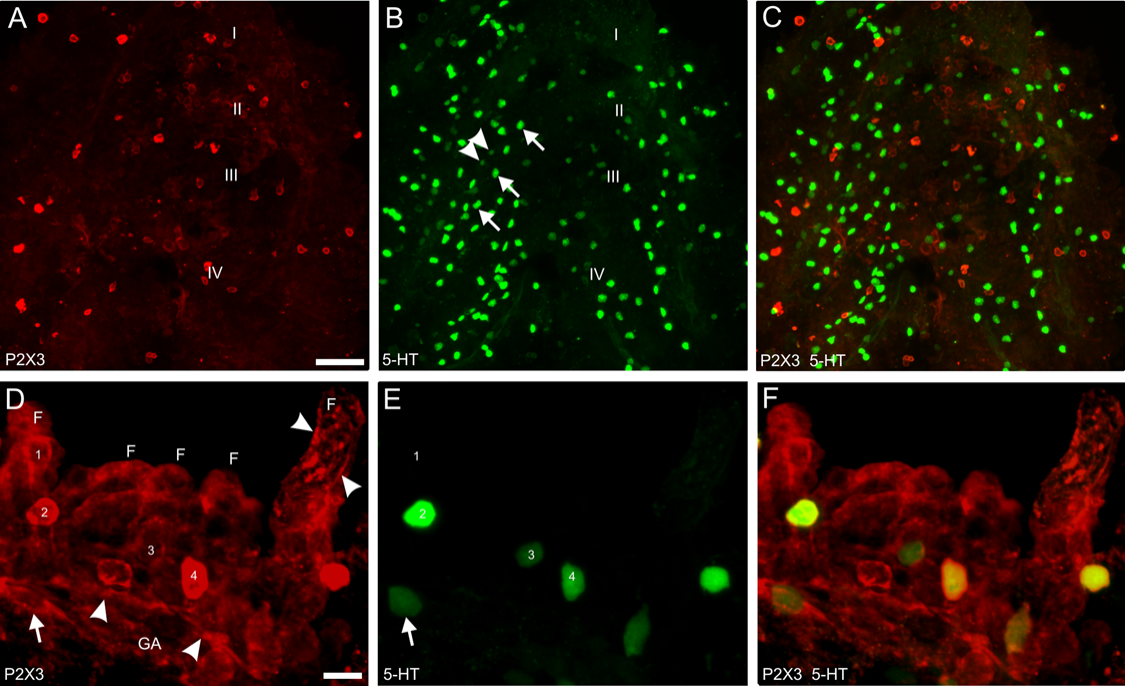
Immunohistochemical localization of P2X3 receptors in the zebrafish gill at 16 days post-fertilization. Co-labelling with antibodies against P2X3 (red) and serotonin (5-HT, green) are shown at low magnification in a whole gill complex in ventral view (A−C), and at higher magnification in an isolated gill arch (D−F). (A) Many P2X3-positive cells were observed on all four gill arches, as indicated by numerals I−IV on the left side of the gill complex. (B) Labelling by anti-5-HT identified primarily Merkel-like cells (arrows) of the gill arches with few neuroepithelial cells (NECs, arrowheads) of the gill filaments visible. (C) Merge of panels A and B. (D) In isolated gills, the gill arch (GA) displayed P2X3-positive neurons (arrow), and a nerve bundle (arrowheads); gill filament primordia (F) retain several P2X3-positive cells (numbered 1−4; note that cell 3 is P2X3-negative). In addition, the filament at the right displayed a pattern of P2X3 activity that was reminiscent of nerve fibres (arrowhead). (E) 5-HT immunoreactivity revealed that P2X3-positive neurons were also 5-HT positive. From D, cell 1 was 5-HT-negative; cells 2 and 4 were P2X3- and 5-HT-positive; cell 3 was 5-HT-positive and P2X3-negative. (F) Merge of panels D and E. Scale bar in A = 50 um; in B = 10 μm.

Based on the effects of suramin and A-317491 upon *f*_V_, we characterized the distribution of P2X3 receptors in the gills using immunohistochemistry (Fig 7). In 21 specimens at 16 dpf, P2X3-immunoreactive cells were found throughout the gill complex. In ventral view, large P2X3 cells were observed in the gill arches among other cell types, such as Merkel-like cells of the gill arches (see [37]) and NECs of the gill filaments, as labelled with anti-5-HT (Fig 7A–7C). Cellular co-localization of P2X3 and 5-HT was not obvious in the tissue from ventral view due to the orientation of the gill filaments, but was clear when gill arches were separated by manipulation and observed at higher magnification (Fig 7D–7F). In the gill arches, P2X3 was localized in 5-HT-positive neurons (Fig 7D and 7E) and was also found in nerve bundles (Fig 7D). In the developing gill filaments P2X3 co-localized with 5-HT in NECs; and P2X3 was also observed in cells and nerve fibres of the filaments that were negative for 5-HT (Fig 7D–7F). Moreover, there were 5-HT-positive NECs that did not express P2X3 (Fig 7D and 7E).

## Discussion

The present study has shown that chemicals with potential effects upon the respiratory response to hypoxia can be rapidly screened in zebrafish at multiple concentrations to generate an activity profile. Our results further demonstrate that purinergic and nicotinic receptors in the gills contribute to the chemosensory response to hypoxia in zebrafish.

### Development of ventilation frequency assays for chemical screening

We have demonstrated a rapid approach to identifying chemical agents that evoke hyperventilation in zebrafish, or that inhibit the hyperventilatory response evoked by co-administration of hypoxia. Previous studies in zebrafish utilized flow-through chambers designed to record the ventilatory response of a single immobilized animal during each trial [23,24,28,38–40]. In the present work, we have made use of multi-well plates to simultaneously treat a greater number of animals, thereby increasing the number of samples per trial. These procedures produced data that conformed to a normal distribution and allowed for parametric statistical testing, rapid generation of dose-response curves for each drug, and calculation of EC_50_. The use of multi-well plates eliminated the need for production of hypoxia by solution exchange within the chamber, which is otherwise time consuming and requires firm immobilization of the specimen to the chamber bottom. Brief placement of multi-well plates in a hypoxic incubator quickly resulted in equilibration of each well to a desired hypoxic *P*_O2_ within the incubator and produced hypoxic ventilatory rates in zebrafish equivalent to those previously reported at the stages tested [23].

In order to determine how readily exogenously-applied chemicals would diffuse across the gill and affect sensory nerve terminals or NECs, we used 5-HT, an abundant neurotransmitter in gill NECs known to elicit hyperventilation [13,14,23,35,36], as a positive control. 5-HT increased *f*_V_ in zebrafish at concentrations from 1−100 μM with an EC_50_ of 14.6 μM. This concentration range approximately corresponds with mammalian data, where 5-HT elicited responses at 1−50 μM in cells dissociated from the rat carotid body [41,42], and at 5−150 μM in O_2_-sensitive neuroepithelial bodies in lung slices from neonatal hamster [43]. One limitation to whole-organism chemical screening, however, is that the effects of each compound on post-synaptic terminals or chemoreceptive NECs of the gills may not be easily dissociated from additional non-specific effects outside of the gills, such as on the central nervous system, that may indirectly affect the ventilatory response. However, the blood-brain barrier in zebrafish develops at approximately 3 dpf [44], suggesting that at such low concentrations the effects of exogenously administered chemicals upon the central nervous system may have been limited in our experiments.

### Neurochemical basis of O_2_ sensing in the gill

The neurochemical mechanisms that underlie O_2_ sensing in the gill are not yet resolved. NECs, or NEC-like cells, containing 5-HT or a vesicular transporter for ACh have been identified in the gills [13,14,21,23]; however, neither the release of 5-HT nor ACh has been observed during a hypoxic challenge. In zebrafish, recent studies investigating the effects of exogenous drug application *in vivo* have shown that the hyperventilatory response to hypoxia is regulated by metabotropic 5-HT2 receptors, ionotropic 5-HT3 receptors, and dopaminergic receptors [23,27]; and that nitric oxide may play a stimulatory or inhibitory role, depending on developmental stage [24]. Earlier studies in the rainbow trout (*Oncorhynchus mykiss*) showed that, compared to ACh, 5-HT induced relatively minor hyperventilation and chemosensory discharge in the gill nerves in the rainbow trout [35,36]. Together these data suggest that hypoxic hyperventilation is regulated by multiple excitatory, inhibitory or modulatory neurotransmitters in the gill.

In type I cells of the mammalian carotid body, the homologues of gill NECs, the primary excitatory neurotransmitters that activate afferent nerve terminals in the carotid body include ACh and ATP [6–11]. In trout, ACh and nicotine produced pronounced sensory discharge in the gill and hyperventilation [35,36]; whilst muscarine and atropine had only minor effects. In zebrafish, preliminary evidence demonstrated that application of the cholinergic agonist, nicotine, increased *f*_V_ [27]; whilst the nicotinic antagonist, hexamethonium, inhibited the hyperventilatory response to hypoxia [23]. The present work agrees with these findings and found that nicotine produced a significant and dose-dependent elevation of *f*_V_; and that atropine was without significant effect, inhibiting the hypoxic response only at high concentration.

Previous studies had not tested whether purinergic drugs might affect *f*_V_ in fish. In the present study, we used suramin, a broad-spectrum antagonist of purinergic P2 receptors, as well as an additional antagonist, A-317491, with specificity for P2X3 and P2X2/3 receptors. Both drugs showed concentration-dependent effects, eliminated the hyperventilatory response to hypoxia, and further reduced *f*_V_ below resting levels. Moreover, A-317491 had a lower EC_50_ than suramin. Administration of the ATP analogue and P2 agonist, 2-MeATP, had limited effects across a range of concentrations, perhaps due to poor diffusion of this drug across the gill epithelium, but successfully induced hyperventilation at 100 μM. It was thus apparent from our experiments that endogenous release of ATP, as during hypoxia, was more effective in producing hyperventilation than exogenous application of the ATP analogue. We confirmed these observations of purinergic activity from behavioural assays by localizing P2X3 receptors to the gills of zebrafish larvae using immunohistochemistry. A previous study found P2X3 labelling in the gill epithelium of adult zebrafish [14]. In the present study, we determined that P2X3 receptors were found in serotonergic neurons of the gill arches. In adult zebrafish, serotonergic neurons reside in the proximal aspect of each gill filament and extend axons that innervate chemoreceptive NECs [14]. In the developing gills of larvae, however, these neurons are observed in the gill arches and appear to migrate to the base of the filaments as the gill matures and increases in size [28]. We suggest that P2X3/5-HT-positive neurons of the gill arches in larvae innervate the NECs of the filaments and provide an afferent pathway for initiation of autonomic reflexes, such as hyperventilation, during hypoxic stimulation of NECs. Indeed, in the present study P2X3 also labelled nerve bundles of the gill arch and generated a labelling pattern in the gill filaments that resembled that of innervation in developing zebrafish [28].

Our present and previous studies [23] suggest a role for ACh via nicotinic receptors, and ATP via P2X3 and possibly other purinergic receptors, as potential neurotransmitters in gill O_2_ sensing in zebrafish. Most compelling among these data is that the hyperventilatory response to hypoxia was inhibited by antagonists of nicotinic receptors [23] and of purinergic receptors, perhaps due to the endogenous release of ACh and ATP, respectively, elicited by hypoxia. These results are in line with evidence from the mammalian carotid body, where both nicotinic and P2X2/3 receptors are found at post-synaptic terminals of petrosal neurons and play critical roles in chemoexcitation during hypoxia [6,8,9–11,45]. We also localized P2X3 activity within 5-HT-positive NECs of the filaments, as well as in other non-serotonergic cells. These observations may indicate a paracrine role for P2X3 receptors within NECs in modulating neurotransmitter release in the gill. For example, in the carotid body "ATP-induced ATP release" involving purinergic P2Y2-expressing glial-like cells can increase the excitatory ATP signal at the chemosensory synapse [46]. In addition, P2X3 expression in NECs may form part of an autocrine, or positive feedback, pathway in which ATP release may be further potentiated. Such a feedback mechanism involving P2X receptors has been observed in taste receptor cells [47]; and P2X2 receptors were localized to carotid body type I cells in rat following exposure to chronic hypoxia [48].

### Conclusions and applications

In this article, we have presented evidence for the novel action of purinergic drugs upon the ventilatory response to acute hypoxia in zebrafish. Our studies suggest that a role for ACh and ATP as neurotransmitters in peripheral O_2_ chemosensing may not be limited to the carotid body in mammals. Furthermore, such a mechanism may have arisen earlier during vertebrate evolution—before the appearance of air-breathing vertebrates. The way in which ACh and ATP may work with 5-HT in the fish gill to coordinate the hypoxic response is unclear, but may involve neurotransmitter release from multiple chemoreceptor populations, and a combination of synaptic, paracrine and autocrine pathways. Exploiting the ventilatory response in zebrafish for chemical screening will help create a neurochemical profile of control of the hypoxic response in this model organism, and may lead to the identification of novel drugs that affect peripheral O_2_-sensing chemoreceptors. By extension, such drugs may be tested subsequently in mammalian systems and developed as therapeutic agents for human disorders involving the carotid body, such as hypertension, chronic heart failure, obstructive sleep apnea, sudden infant death syndrome, and diabetes [5,49,50].

Additionally, results of these studies may contribute to the establishment of new policies aimed at the preservation of water ecosystems. Effluent from industry, pharmaceuticals and household products may have profound effects upon the health of aquatic organisms, including fish. Ammonia, a commercially important chemical, acts directly upon gill NECs and causes hyperventilation in fish [25,51]. Fluoxetine, the selective 5-HT reuptake inhibitor in the antidepressant, Prozac, is found in effluent [52] and is a ventilatory stimulant in rainbow trout [53]. A laboratory screen for the ventilatory effects of chemicals isolated from the environment may establish a procedure for early detection of chemicals that may otherwise have catastrophic effects upon aquatic populations.

## Supporting Information

S1 Fig Illustration of a multi-well plate used in behavioural assays. The first three rows (rows A, B and C) are coated with Sylgard (see Materials and Methods). The well marked "A1" is additionally filled with solution and a zebrafish larva. Scale bar = 16 mm.
(TIF)

S2 Fig Illustration of a single larva immersed in solution in a well of a multi-well plate. The image is the same as in well "A1" in S1 Fig but at higher magnification. The well was previously coated with Sylgard and contains solution. Scale bar = 2 mm.
(TIF)

S3 Fig Preliminary trials determined the optimal duration for exposure of zebrafish larvae to 8 mmHg hypoxia. Mean ± s.e.m. ventilation frequency (*f*_V_, in min^-1^) was measured in normoxic controls (Con) and after application of hypoxia (Hox) for 5 min (A), 7 min (B), and 10 min (C). Asterisks indicate a significant difference from control (n = 10, 7, and 8 in panels A–C; P < 0.05, paired *t*-test). (D) Data taken from the previous panels indicated that 7 min hypoxia produced the greatest increase in *f*_V_ above controls.
(TIFF)

S4 Fig 2-MeSATP increased mean ± s.e.m. ventilation frequency (*f*_V_, in min^-1^). (A−E) Baseline *f*_V_ was measured as control (Con) and 1, 25, 50, 100 or 200 μM 2-MeSATP was applied. Normal solution was replaced during recovery (Rec). Asterisk in D indicates a significant difference from control (*n* = 11, 10, 10, 9 and 10; *P* < 0.05, repeated measures ANOVA-Bonferroni).
(TIFF)

## References

[1] López-Barneo J, López-López JR, Ureña J, González C. Chemotransduction in the carotid body: K^+^ current modulated by P_O2_ in type I chemoreceptor cells. Science. 1988; 241:580–582. 2456613 10.1126/science.2456613

[2] Gonzalez C, Almaraz L, Obeso A, Rigual R. Carotid body chemoreceptors: from natural stimuli to sensory discharges. Physiol Rev. 1994; 74: 829–898. 7938227 10.1152/physrev.1994.74.4.829

[3] Buckler KJ. TASK-like potassium channels and oxygen sensing in the carotid body. Respir Physiol Neurobiol. 2007; 157: 55–64. 17416212 10.1016/j.resp.2007.02.013

[4] Peers C, Wyatt CN, Evans AM. Mechanisms for acute oxygen sensing in the carotid body. Respir Physiol Neurobiol. 2010; 174: 292–298. 10.1016/j.resp.2010.08.010 20736087

[5] Kumar P, Prabhakar NR. Peripheral chemoreceptors: function and plasticity of the carotid body. Compr Physiol. 2012; 2:141–219. 10.1002/cphy.c100069 23728973 PMC3919066

[6] Nurse CA, Zhang M. Acetylcholine contributes to hypoxic chemotransmission in co-cultures of rat type 1 cells and petrosal neurons. Respir Physiol. 1999; 115: 189–199. 10385033 10.1016/s0034-5687(99)00017-1

[7] Zhang M, Zhong H, Vollmer C, Nurse CA. Co-release of ATP and ACh mediates hypoxic signalling at rat carotid body chemoreceptors. J Physiol. 2000; 525: 143–158. 10811733 10.1111/j.1469-7793.2000.t01-1-00143.x PMC2269919

[8] Iturriaga R, Alcayaga J. Neurotransmission in the carotid body: transmitters and modulators between glomus cells and petrosal ganglion nerve terminals. Brain Res Brain Res Rev. 2004; 47: 46–53. 15572162 10.1016/j.brainresrev.2004.05.007

[9] Shirahata M, Balbir A, Otsubo T, Fitzgerald RS. Role of acetylcholine in neurotransmission of the carotid body. Respir Physiol Neurobiol. 2007;157: 93–105. 17284361 10.1016/j.resp.2006.12.010

[10] Fitzgerald RS, Shirahata M, Chang I, Kostuk E. The impact of hypoxia and low glucose on the release of acetylcholine and ATP from the incubated cat carotid body. Brain Res. 2009; 1270: 39–44. 10.1016/j.brainres.2009.02.078 19285968

[11] Nurse CA. Neurotransmitter and neuromodulatory mechanisms at peripheral arterial chemoreceptors. Exp Physiol. 2010; 95: 657–667. 10.1113/expphysiol.2009.049312 20360424

[12] Kahlin J, Mkrtchian S, Ebberyd A, Hammarstedt-Nordenvall L, Nordlander B, Yoshitake T, et al The human carotid body releases acetylcholine, ATP and cytokines during hypoxia. Exp Physiol. 2014; 99: 1089–1098. 10.1113/expphysiol.2014.078873 24887113

[13] Dunel-Erb S, Bailly Y, Laurent P. Neuroepithelial cells in fish gill primary lamellae. J Appl Physiol Respir Environ Exerc Physiol. 1982; 53: 1342–1353. 7153134 10.1152/jappl.1982.53.6.1342

[14] Jonz MG, Nurse CA. Neuroepithelial cells and associated innervation of the zebrafish gill: a confocal immunofluorescence study. J Comp Neurol. 2003; 461: 1–17. 12722101 10.1002/cne.10680

[15] Milsom WK, Burleson ML. Peripheral arterial chemoreceptors and the evolution of the carotid body. Respir Physiol Neurobiol. 2007; 157: 4–11. 17353155 10.1016/j.resp.2007.02.007

[16] Jonz MG. Oxygen Sensing In: Evans DH, Claiborne JB, Currie S, editors. The Physiology of Fishes, 4th ed. Boca Raton CRC Press; 2014 pp. 149–175.

[17] Jonz MG, Fearon IM, Nurse CA. Neuroepithelial oxygen chemoreceptors of the zebrafish gill. J Physiol. 2004; 560:737–752. 15331683 10.1113/jphysiol.2004.069294 PMC1665292

[18] Burleson ML, Mercer SE, Wilk-Blaszczak MA. Isolation and characterization of putative O_2_ chemoreceptor cells from the gills of channel catfish (*Ictalurus punctatus*). Brain Res. 2006; 1092: 100–107. 16690040 10.1016/j.brainres.2006.03.085

[19] Perry SF, Jonz MG, Gilmour KM. Oxygen Sensing and the Hypoxic Ventilatory Response In: Richards J, Farrell A, Brauner C, editors. Hypoxia. Waltham: Academic Press 2009 pp. 193–253.

[20] Milsom WK. New insights into gill chemoreception: receptor distribution and roles in water and air breathing fish. Respir Physiol Neurobiol. 2012; 184: 326–339. 10.1016/j.resp.2012.07.013 22841952

[21] Regan KS, Jonz MG, Wright PA. Neuroepithelial cells and the hypoxia emersion response in the amphibious fish *Kryptolebias marmoratus*. J Exp Biol. 2011; 214: 2560–2568. 10.1242/jeb.056333 21753050

[22] Porteus CS, Brink DL, Milsom WK. Neurotransmitter profiles in fish gills: putative gill oxygen chemoreceptors. Respir Physiol Neurobiol. 2012; 184: 316–325. 10.1016/j.resp.2012.06.019 22728948

[23] Shakarchi K, Zachar PC, Jonz MG. Serotonergic and cholinergic elements elements of the hypoxic ventilatory response in devloping zebrafish. J Exp Biol. 2013; 216: 869–880. 10.1242/jeb.079657 23155078

[24] Porteus CS, Pollack J, Tzaneva V, Kwong RW, Kumai Y, Abdallah SJ, et al A role for nitric oxide in the control of breathing in zebrafish (*Danio rerio*). J Exp Biol. 2015; In press. 10.1242/jeb.127795 26486367

[25] Zhang L, Nurse CA, Jonz MG, Wood CM. Ammonia sensing by neuroepithelial cells and ventilatory responses to ammonia in rainbow trout. J Exp Biol. 2011; 214: 2678–2689. 10.1242/jeb.055541 21795563

[26] Abdallah SJ, Jonz MG, Perry SF. Extracellular H^+^ induces Ca^2+^ signals in respiratory chemoreceptors of zebrafish. Pflugers Arch. 2015; 467: 399–413. 10.1007/s00424-014-1514-2 24770973

[27] Jonz MG, Zachar PC, Da Fonte DF, Mierzwa AS. Peripheral chemoreceptors in fish: a brief history and a look ahead. Comp Biochem Physiol PartA. 2015; 186: 27–38. 10.1016/j.cbpa.2014.09.002 25218943

[28] Jonz MG, Nurse CA. Development of oxygen sensing in the gills of zebrafish. J Exp Biol. 2005; 208: 1537–1549. 15802677 10.1242/jeb.01564

[29] Tamplin OJ, White RM, Jing L, Kaufman CK, Lacadie SA, Li P, et al Small molecule screening in zebrafish: swimming in potential drug therapies. Wiley Interdiscip Rev Dev Biol. 2012; 1:459–468. 10.1002/wdev.37 23801494 PMC8904173

[30] Rennekamp AJ, Peterson RT. 15 years of zebrafish chemical screening. Curr Opin Chem Biol. 2015; 24: 58–70. 10.1016/j.cbpa.2014.10.025 25461724 PMC4339096

[31] Westerfield M. The Zebrafish Book A Guide for the Laboratory Use of Zebrafish (Danio rerio), 4th ed. Eugene: University of Oregon Press; 2000.

[32] Rombough PJ. Gills are needed for ionoregulation before they are needed for O_2_ uptake in developing zebrafish, *Danio rerio*. J Exp Biol. 2002; 205: 1787–1794. 12042337 10.1242/jeb.205.12.1787

[33] Kokel D, Peterson RT. Using the zebrafish photomotor response for psychotropic drug screening. Methods Cell Biol. 2011; 105: 517–24. 10.1016/B978-0-12-381320-6.00022-9 21951545 PMC3635141

[34] Saltys HA, Jonz MG, Nurse CA. Comparative study of gill neuroepithelial cells and their innervation in teleosts and *Xenopus* tadpoles. Cell Tissue Res. 2006; 323: 1–10. 16163489 10.1007/s00441-005-0048-5

[35] Burleson ML, Milsom WK. Cardio-ventilatory control in rainbow trout: I. Pharmacology of branchial, oxygen-sensitive chemoreceptors. Respir Physiol. 1995; 100: 231–238. 7481112 10.1016/0034-5687(95)91595-x

[36] Burleson ML, Milsom WK. Cardio-ventilatory control in rainbow trout: II. Reflex effects of exogenous neurochemicals. Respir Physiol. 1995; 101: 289–299. 10.1016/0034-5687(95)00029-d 8607001

[37] Zachar PC, Jonz MG. Confocal imaging of Merkel-like basal cells in the taste buds of zebrafish. Acta Histochem. 2012; 114: 101–115 10.1016/j.acthis.2011.03.006 21477848

[38] Turesson J, Schwerte T, Sundin L. Late onset of NMDA receptor-mediated ventilatory control during early development in zebrafish (*Danio rerio*). Comp Biochem Physiol A Mol Integr Physiol. 2006;143: 332–339. 16458555 10.1016/j.cbpa.2005.12.008

[39] Zhang D, Xi Y, Coccimiglio ML, Mennigen JA, Jonz MG, Ekker M, et al Functional prediction and physiological characterization of a novel short trans-membrane protein 1 as a subunit of mitochondrial respiratory complexes. Physiol Genomics. 2012; 44: 1133–1140. 10.1152/physiolgenomics.00079.2012 23073385

[40] Porteus CS, Abdallah SJ, Pollack J, Kumai Y, Kwong RW, Yew HM, et al The role of hydrogen sulphide in the control of breathing in hypoxic zebrafish (*Danio rerio*). J Physiol. 2014; 592: 3075–3088. 10.1113/jphysiol.2014.271098 24756639 PMC4214661

[41] Zhang M, Nurse CA. Does endogenous 5-HT mediate spontaneous rhythmic activity in chemoreceptor clusters of rat carotid body? Brain Res. 2000; 872: 199–203. 10924693 10.1016/s0006-8993(00)02499-9

[42] Zhang M, Fearon IM, Zhong H, Nurse CA. Presynaptic modulation of rat arterial chemoreceptor function by 5-HT: role of K^+^ channel inhibition via protein kinase C. J Physiol. 2003; 551: 825–842. 12826651 10.1113/jphysiol.2002.038489 PMC2343291

[43] Fu XW, Wang D, Pan J, Farragher SM, Wong V, Cutz E. Neuroepithelial bodies in mammalian lung express functional serotonin type 3 receptor. Am J Physiol Lung Cell Mol Physiol. 2001; 281: L931–940. 11557597 10.1152/ajplung.2001.281.4.L931

[44] Xie J, Farage E, Sugimoto M, Anand-Apte B. A novel transgenic zebrafish model for blood-brain and blood-retinal barrier development. BMC Dev Biol. 2010; 10: 76 10.1186/1471-213X-10-76 20653957 PMC2914679

[45] Nurse CA, Piskuric NA. Signal processing at mammalian carotid body chemoreceptors. Semin Cell Dev Biol. 2013; 24: 22–30. 10.1016/j.semcdb.2012.09.006 23022231

[46] Nurse CA. Synaptic and paracrine mechanisms at carotid body arterial chemoreceptors. J Physiol. 2014; 592: 3419–3426. 10.1113/jphysiol.2013.269829 24665097 PMC4229340

[47] Kinnamon SC, Finger TE. A taste for ATP: neurotransmission in taste buds. Front Cell Neurosci. 2013; 7: 264 10.3389/fncel.2013.00264 24385952 PMC3866518

[48] He L, Chen J, Dinger B, Stensaas L, Fidone S. Effect of chronic hypoxia on purinergic synaptic transmission in rat carotid body. J Appl Physiol. 2006; 100: 157–162. 16357082 10.1152/japplphysiol.00859.2005

[49] Gauda EB, Cristofalo E, Nunez J. Peripheral arterial chemoreceptors and sudden infant death syndrome. Respir Physiol Neurobiol. 2007; 157: 162–170. 17446144 10.1016/j.resp.2007.02.016

[50] Gao L, Ortega-Sáenz P, García-Fernández M, González-Rodríguez P, Caballero-Eraso C, López-Barneo J. Glucose sensing by carotid body glomus cells: potential implications in disease. Front Physiol. 2014; 5: 398 10.3389/fphys.2014.00398 25360117 PMC4197775

[51] Zhang L, Wood CM. Ammonia as a stimulant to ventilation in rainbow trout *Oncorhynchus mykiss*. Respir Physiol Neurobiol. 2009; 168: 261–271. 10.1016/j.resp.2009.07.011 19619676

[52] Brooks BW, Turner PK, Stanley JK, Weston JJ, Glidewell EA, Foran CM, et al Waterborne and sediment toxicity of fluoxetine to select organisms. Chemosphere. 2003; 52: 135–142. 12729696 10.1016/S0045-6535(03)00103-6

[53] Kermorgant M, Lancien F, Mimassi N, Tyler CR, Le Mével JC. Effects of intracerebroventricular administered fluoxetine on cardio-ventilatory functions in rainbow trout (*Oncorhynchus mykiss*). Gen Comp Endocrinol. 2014; 205: 176–184. 10.1016/j.ygcen.2014.03.012 24681193

