# Supplementary figures and images for: Purinergic and Cholinergic Drugs Mediate Hyperventilation in Zebrafish: Evidence from a Novel Chemical Screen
Source: PLoS One. 2016 Apr 21;11(4):e0154261. doi: 10.1371/journal.pone.0154261 (PMC4839714; doi:10.1371/journal.pone.0154261)

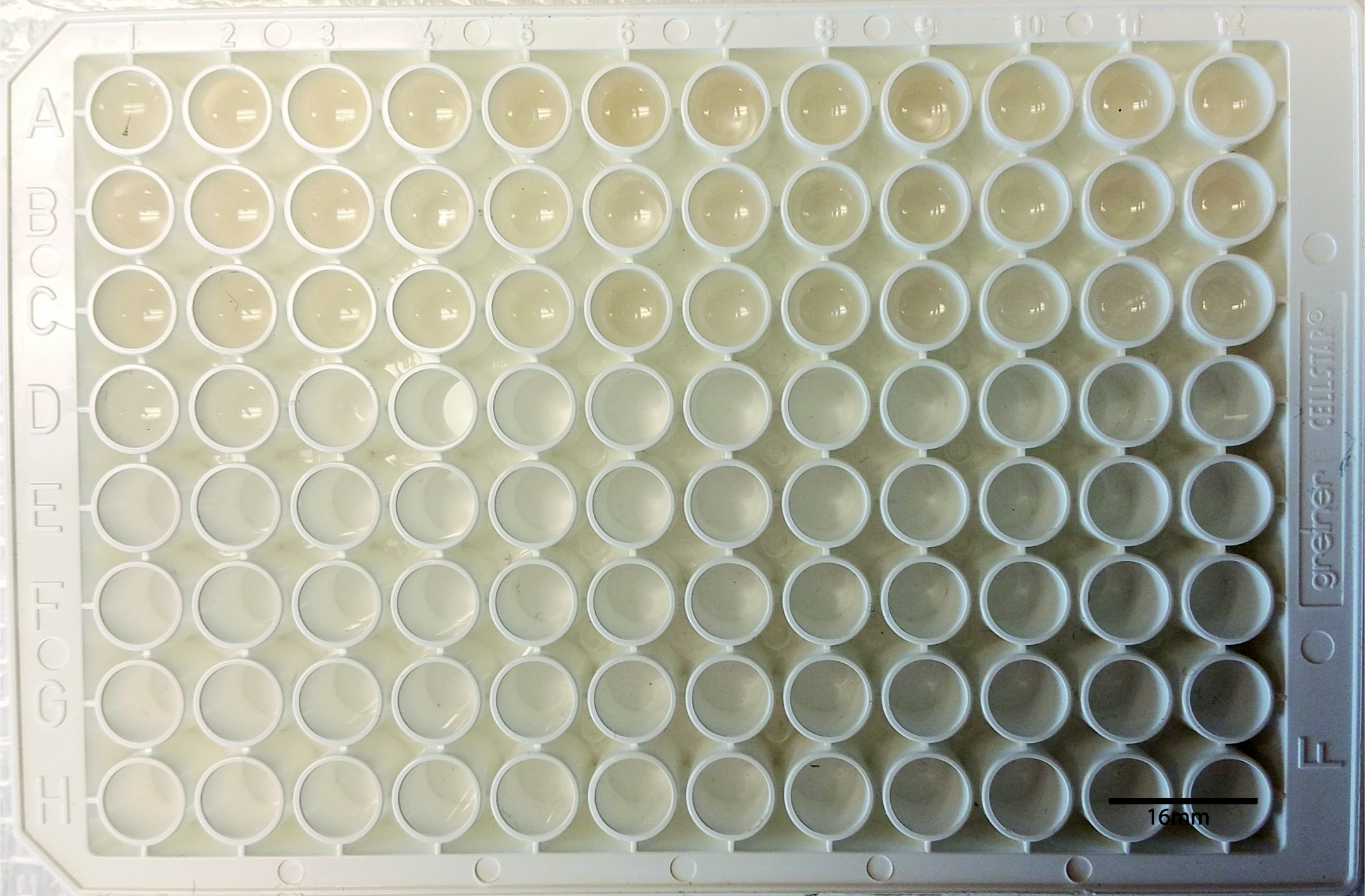

Supplement: S1 Fig — The first three rows (rows A, B and C) are coated with Sylgard (see Materials and Methods). The well marked "A1" is additionally filled with solution and a zebrafish larva. Scale bar = 16 mm. (TIF) [file pone.0154261.s001.tif]

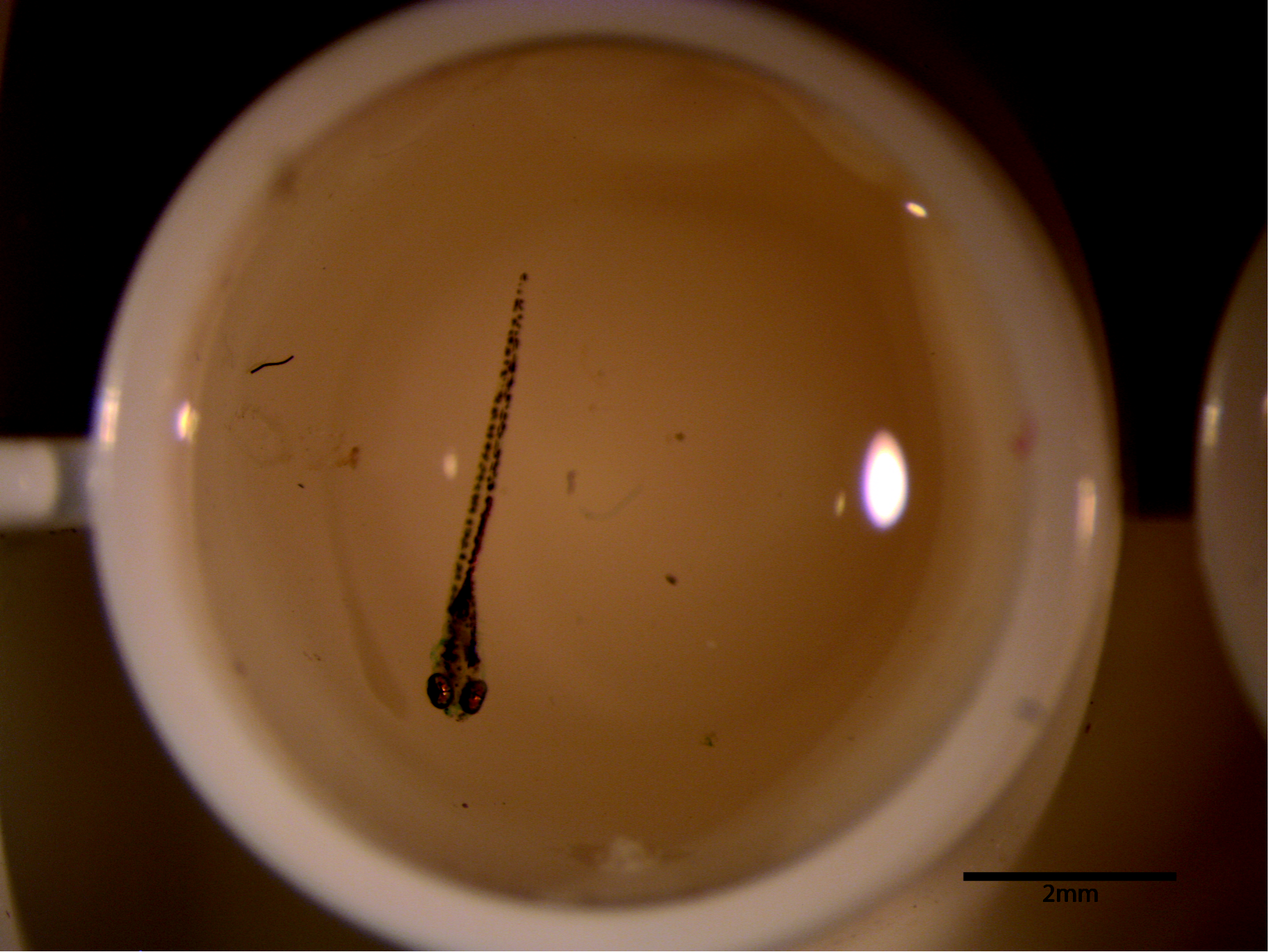

Supplement: S2 Fig — The image is the same as in well "A1" in S1 Fig but at higher magnification. The well was previously coated with Sylgard and contains solution. Scale bar = 2 mm. (TIF) [file pone.0154261.s002.tif]

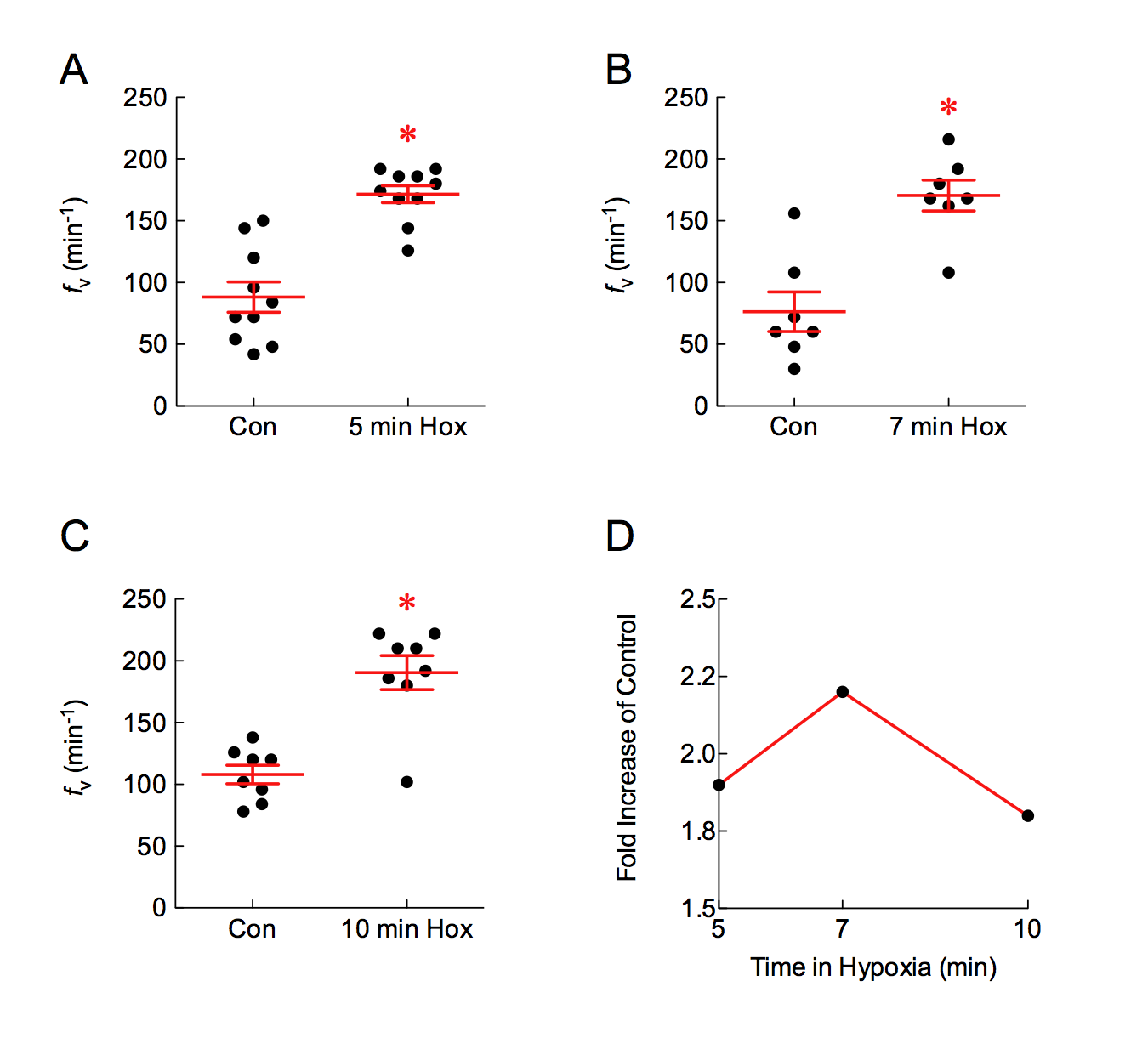

Supplement: S3 Fig — Mean ± s.e.m. ventilation frequency (fV, in min-1) was measured in normoxic controls (Con) and after application of hypoxia (Hox) for 5 min (A), 7 min (B), and 10 min (C). Asterisks indicate a significant difference from control (n = 10, 7, and 8 in panels A–C; P < 0.05, paired t-test). (D) Data taken from the previous panels indicated that 7 min hypoxia produced the greatest increase in fV above controls. (TIFF) [file pone.0154261.s003.tiff]

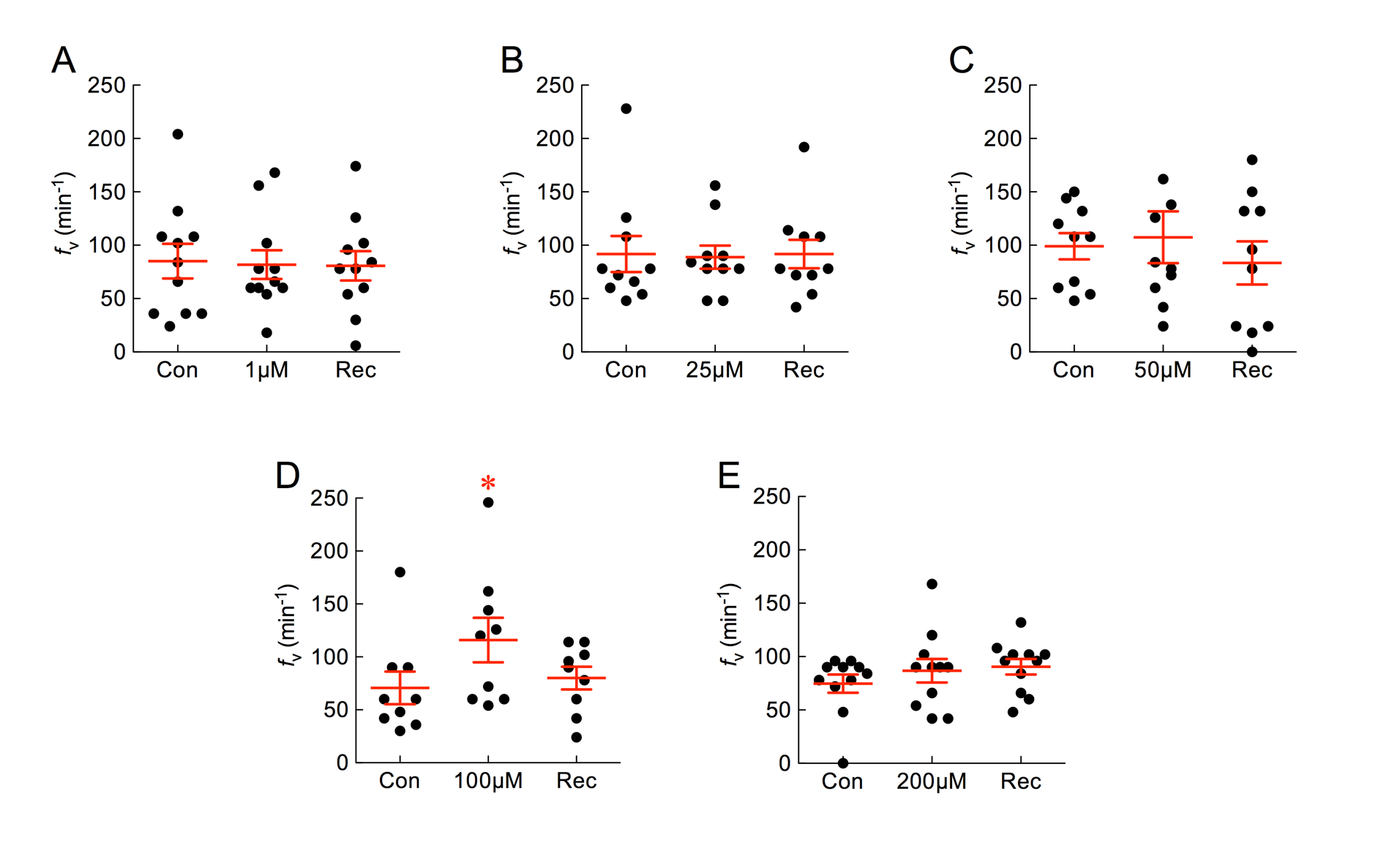

Supplement: S4 Fig — (A−E) Baseline fV was measured as control (Con) and 1, 25, 50, 100 or 200 μM 2-MeSATP was applied. Normal solution was replaced during recovery (Rec). Asterisk in D indicates a significant difference from control (n = 11, 10, 10, 9 and 10; P < 0.05, repeated measures ANOVA-Bonferroni). (TIFF) [file pone.0154261.s004.tiff]
